# Extraction Bottleneck in the Diagnosis of SARS-CoV-2: Evaluation of an Alternative Protocol Derived from Veterinary Use

**DOI:** 10.3390/microorganisms11020535

**Published:** 2023-02-20

**Authors:** Paolo Bottino, Elisa Zanotto, Francesca Sidoti, Lisa Pastrone, Roberto Piva, Elisabetta Mereu, Cristina Costa, Rossana Cavallo

**Affiliations:** 1 Microbiology and Virology Unit, A.O.U. “Città della Salute e della Scienza di Torino”, 10126 Turin, Italy; 2 Department of Molecular Biotechnology and Health Sciences, University of Turin, 10126 Turin, Italy

**Keywords:** SARS-CoV-2, COVID-19, automated extraction, RNA, liquid handler

## Abstract

The COVID-19 pandemic represented a challenge for health-care systems, and a major bottleneck in SARS-CoV-2 diagnosis was the unavailability of extraction reagents. To overcome this limitation, we performed a comparative analysis to evaluate the performance of an alternative extraction protocol derived from veterinary use adapted to an open robotic platform (Testing method). A total of 73 nasopharyngeal swabs collected for diagnosis of SARS-CoV-2 infection were simultaneously extracted with the Testing protocol and the laboratory Standard of Care in order to assess the performance of the first one. The Cohen’s coefficient between both procedures was excellent (K Value = 0.955). Analysis of cycle threshold and linear regression showed a significant correlation between the two methods for each tested genetic target. Although validated for veterinary applications, the Testing method showed excellent performances in RNA extraction, with several advantages: lower sample input volume, the possibility to overcome the lack of deep-well plates and adaptability to robotic liquid handlers.

## 1. Introduction

Coronavirus disease 2019 (COVID-19) is a viral disease caused by severe acute respiratory syndrome coronavirus 2 (SARS-CoV-2), a novel betacoronavirus originally reported in patients with pneumonia by some local Chinese health facilities in December 2019 [1]. Most of the patients were linked with the Huanan Seafood Wholesale market in Jianghan District, Wuhan [2]. SARS-CoV-2 is transmitted among the human population through respiratory droplets, aerosol and direct or indirect contact. Moreover, the presence of asymptomatic and pre-symptomatic infected persons might also be a potential source of infection explaining the sudden increase of the virus [3,4]. Due to rapid spread and severity of disease, the increase in cases outside China and the number of affected countries, the WHO declared the SARS-CoV-2 infection a global pandemic in March 2020 [5].

The diagnosis of COVID-19 is made by qualitative Reverse Transcription qPCR (RT-qPCR) testing of specimens collected at a level of the upper respiratory tract (nasopharyngeal and oropharyngeal secretion) [6]. Quick and accurate detection of virus-infected individuals is important to minimize the spread of SARS-CoV-2, and it immediately represented a substantial challenge for healthcare systems. Different detection protocols have been released early in the pandemic by CDC-China, Pasteur Institute-France, CDC-USA, NIID-Japan, Charité-Germany, HKU-Hong Kong and NIH-Thailand, following by the development of more accurate commercial RT-qPCR kits by different manufacturers [7,8]. Although they provided relatively rapid results (average 3–4 h), several problems emerged in clinic diagnostics, particularly in RNA isolation from clinical samples [9]. This step is pivotal since both quality and quantity of the genome obtained could affect the subsequent amplification process [10]. RNA isolation procedures typically involve three general steps: cell lysis, separation of RNA from other macromolecules such as DNA, proteins and lipids and the following RNA concentration; however, it was a procedure that wasted time and resources [11]. Especially the lack of extraction reagents and consumables due to the increasing number of requests for SARS-CoV-2 testing was the major bottleneck in the diagnostic process; therefore, different alternative homemade procedures were evaluated [12,13,14]. Moreover, in order to improve testing throughput, different reports suggested the successful adaptation of generic in house or commercial bead-based protocols on open-deck liquid handling instruments [15].

In order to overcome the unavailability of extraction reagents, the present study is aimed at evaluating the effectiveness of SARS-CoV-2 RNA detection from nasopharyngeal swabs using a modified protocol derived from veterinary use with the MagMAX™-96 AI/ND Viral RNA Isolation Kit (Thermo Fisher Scientific, Waltham, MA, USA) adapted to a robotic open platform StarLet (Hamilton Company, Reno, NV, USA).

## 2. Materials and Methods

### 2.1. Study Design

The study was performed on a total of 73 nasopharyngeal swabs collected and stored at Microbiology and Virology Laboratory of A.O.U. “Città della Salute e della Scienza di Torino” (Turin, Italy) for diagnosis of SARS-CoV-2 infection. All samples were processed according to WHO guidelines [16] and collected in 3 mL of liquid Universal Transport Medium (UTM) (Copan, Italy).

### 2.2. Total RNA Extraction

**Figure 1:**
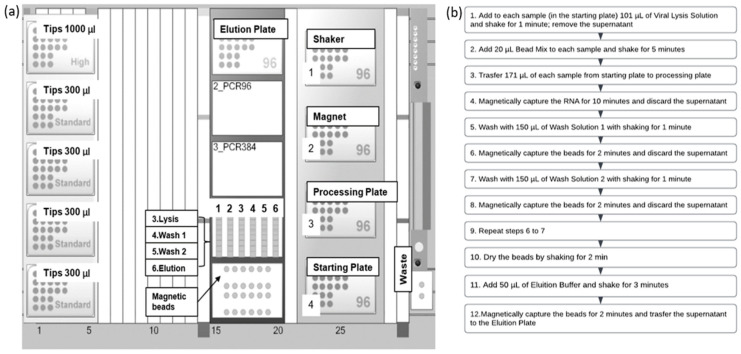
Details of automated extraction protocol: (**a**) set-up of working desk, (**b**) extraction protocol.

For Standard of Care (SoC) analysis (hereinafter named as Reference method), total RNA extraction was performed using an EZ1^®^ Advanced XL instrument (Qiagen, Hilden, Germany) with EZ1^®^ DSP Virus Kit (Qiagen, Hilden, Germany) according to manufacturer instructions: starting volume was 200 µL, while elution volume was 90 µL. Instead, for the MagMAX™-96 AI/ND Viral RNA Isolation kit adapted to automatic platform Hamilton StarLet (hereinafter named as Testing method), 50 µL of each sample were added to a 96 well plate with U bottom (starting plate), and then the reactive was resuspended according to kit instructions. Afterwards, each component was placed on working desk (Figure 1a), and extraction protocol was performed as reported in Figure 1b.

### 2.3. RT-qPCR

Amplification was performed on Bio-Rad CFX 96 Thermal Cycler (Bio-Rad Laboratories, Inc., Hercules, CA, USA) with Seegene Allplex™ SARS-CoV-2 Assay kit (Seegene Inc., Seoul, Republic of Korea) to detect SARS-CoV-2 RNA. According to the manufacturer instructions the cycling conditions were 50 °C for 20 min (reverse transcription), followed by 95 °C for 15 min (initial denaturation), 45 cycles of 95 °C for 10 s (denaturation), 60 °C for 15 s (annealing) and 72 °C for 10 s (extension). The detected targets were E (FAM Channel), N (Quasar 670 channel), RdRP (Cal Red 610 channel) and Internal Control (HEX channel), and samples were considered (tested) positive for SARS-CoV-2 infection if at least one target was detected. Data collection was performed with software SARS-CoV-2 Viewer Version 3.19.001.002 (Seegene Inc., Seoul, Republic of Korea).

### 2.4. Data Analysis

The overall agreement for the Testing method towards the Reference method was performed in terms of sensitivity and specificity. Performance of the Testing method was evaluated also in terms of Cycle Threshold difference (ΔCt) and K-cohen Index compared to the Standard of Care. Discrepancies between the two methods were retested with a third analysis in Transcription Mediated Assay (TMA) with Aptima SARS-CoV-2 Kit (Hologic Inc., Marlborough, MA, USA) on automated platform Panther Plus (Hologic Inc., Marlborough, MA, USA). For each molecular target (E gene, RdRp Gene, N Gene) a linear regression between the two methods was performed. Pearson’s correlation coefficient test was performed to identify the relationship of cycle threshold (Ct) values between the two methods. A *p*-value of 0.05 was the cut-off for statistical significance.

Finally, Limit of Detection (LoD) was tested in triplicate with well-known concentration samples from 1000 to 0.01 TCID 50/mL.

## 3. Results

### 3.1. Clinical Performance

To investigate the clinical performance of the Testing method toward the Reference method, a total of 73 samples were tested: 59 of them tested positive for both the Reference method and the testing one; only one sample was detected positive exclusively by the Reference method. Specifically, all three genetic targets (E, RdRp, N) were detected in 56 samples for both methods, while similarly two samples resulted positive for two targets, and one sample only for one (N or RdRp) (Table 1). When retested with a TMA method, the discrepant specimen confirmed positive with RLU value of 985. Instead, 13 samples tested negative for both methods. Therefore, the overall sensitivity rate for the Testing method was 98.33%, while specificity was 100%. The Cohen’s kappa coefficient between the two methods was excellent (k-value: 0.955) (Table 1), suggesting a high correlation.

### 3.2. Cycle Threshold Analysis

**Figure 2:**
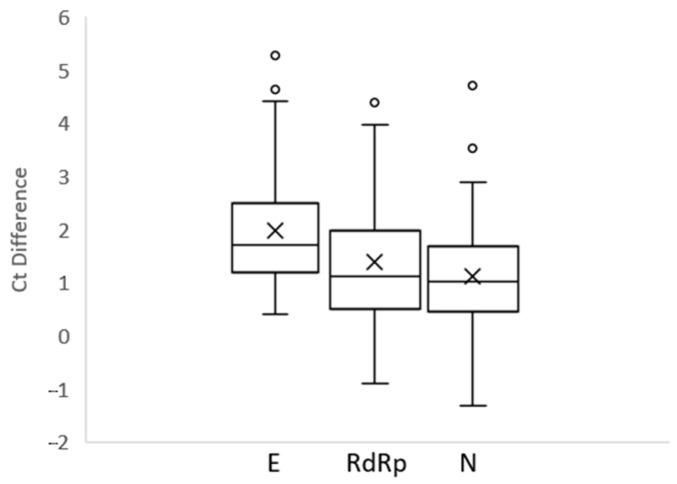
Difference of the cycle threshold (ΔCt) between Reference method and Testing method.

Focusing on cycle threshold difference (ΔCt) between the two extraction methods, results were respectively +1.98 ± 1.17 Ct, +1.39 ± 1.12 Ct and +1.11 ± 1.01 Ct for E, RdRp and N genes, with higher values for the Testing method (Figure 2). These slight differences could be due to different starting extraction volumes (200 µL for the Reference method compared to 50 µL for the Testing method).

**Figure 3:**
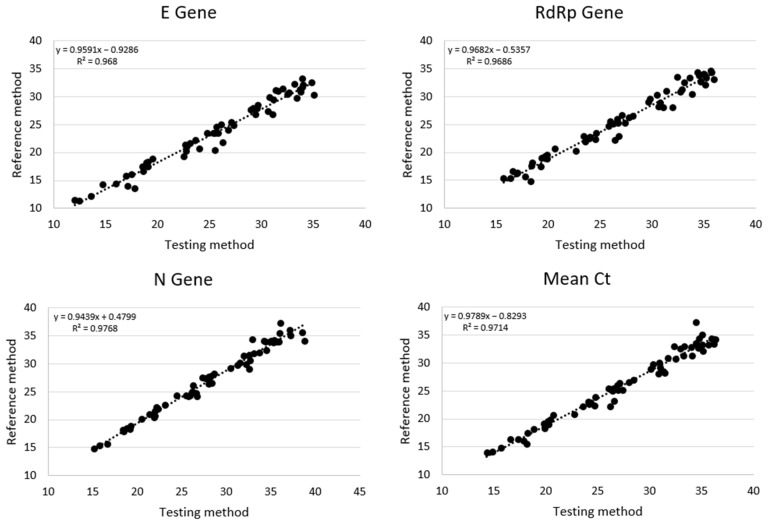
Cycle Threshold correlation between Reference method and Testing method.

The relationship of Ct values for each positive sample between the Reference and Testing method was reported in Figure 3, with a significant strong correlation between the two methods for each genetic target (E gene: R = 0.9839, *p*-value < 0.00001; RdRp gene: R = 0. 9842, *p*-value < 0.00001; N gene: R = 0. 9883, *p*-value < 0.00001). Additionally, the overall correlation with the mean of three genes showed comparable results (R = 0.9856, *p*-value < 0.00001).

### 3.3. Limit of Detection

In order to evaluate the Limit of Detection for both methods, serial dilutions of SARS-CoV-2 samples in UTM were tested in triplicate. All three genes in the range from 1000 TCID50/mL to 0.01 TCID50/mL resulted correctly detected until the lower dilution with both the Reference and the Testing method (Table 2).

Overall, our data suggested a strong correlation between the Reference method and our alternative extraction workflow based on a commercial kit and an automated platform.

## 4. Discussion

The COVID-19 pandemic became the catalyst for the development of accurate detection methods for SARS-CoV-2 in order to better support the clinicians and front-line healthcare professionals. However, development of effective vaccines and the availability of high-quality diagnostic methods remain essential [17,18]. Currently, one of the critical points remains the bottleneck of nucleic acid extraction kits, also due to the spread of the different and more transmissible newest SARS-CoV-2 lineages (BA.4, BA.5, BQ.1, XXB). Various approaches, including reagent-free testing and modification of resources, are currently being developed to overcome these shortages and to increase the ability to test for SARS-CoV-2 [19].

In the present study, we report the clinical performance of our modified automated extraction method when compared with the laboratory Standard of Care. Results showed an excellent agreement between the two tested methods in terms of sensitivity, specificity (Table 1) and Ct relationship (Figure 2 and Figure 3). Indeed, for the Testing method the overall Cohen’s agreement coefficient was high (K value = 0.955). Moreover, evaluation of LoD for both techniques resulted in comparable values despite the different ratio starting volume/elution volume (1:2.2 vs. 1:1, respectively, for the Reference and Testing methods). This suggested the high extraction capacity for our procedure also starting from low volumes of sample combined to versatility of automated robotic platform, in accordance with other studies [12,20,21]. Moreover, the reduction of extraction volumes also provided the possibility to overcome the lack of deep well plates, which were replaced by common 96 well plates with round bottoms to save on the consumption of extraction reagents. Focusing on this latter technical aspect, our work, different from other studies, refs. [12,14,15] highlights the opportunity to use a different type of plate, while for several kits or procedures deep well plates are necessary, also due to the highest extraction volumes required. In our experience, the possibility to adapt several types of 96 well plates (different formats from distinct manufacturers), combined with the reusable reservoirs for extraction reagents, allowed us to overcome limitations due to plastic scarcity.

Limitations related to our study were the small number of samples, and the procedure was tested only on a single automate platform (Hamilton Starlet), starting from nasopharyngeal swabs preserved in UTM medium. More in-depth analysis is required to better evaluate the extraction performance of MagMAX™-96 AI/ND Viral RNA Isolation kit on various matrix (Amies medium, blood, serum, plasma, respiratory samples, for instance) and different open automated robotic liquid handlers.

## 5. Conclusions

Although validated for veterinary applications, our adapted extraction method showed excellent performances in RNA extraction when compared with laboratory SoC, with important advantage to make up for the shortage of plastic and reagents.

## Figures and Tables

**Table 1:** Clinical performances of Testing method and Reference method.

	Testing Method	Reference Method
Positive samples	59	60
3/3 genes	56	57
2/3 genes	2	2
1/3 genes	1	1
Negative samples	13 (+1 *)	13
Total	73	73
Overall Sensitivity	98.33%	
Overall Specificity	100%	
Cohen’s kappa coefficient	0.955 (excellent)	

* The discrepant sample retested with TMA method (Aptima SARS-CoV-2 Kit) resulted positive (Relative Light Unit Value: 985).

**Table 2:** Limit of detection for the Reference method and the Testing method.

Concentration (In TCID_50_/_mL_)	Reference Method			Testing Method		
	Rep 1	Rep 2	Rep 3	Rep 1	Rep 2	Rep 3
1000	+	+	+	+	+	+
100	+	+	+	+	+	+
10	+	+	+	+	+	+
1	+	+	+	+	+ *	+
0.1	+	+	+	+	+	+
0.01	+	+	+	+	+	+

* Only 2/3 genes detected.

## Data Availability

The data contained in this manuscript are available upon request.
